# Green-Synthesized *Leucas aspera*-Functionalized ZnO Nanoparticles for Antibacterial and Anticancer Biomedical Applications

**DOI:** 10.3390/biomimetics11070461

**Published:** 2026-07-02

**Authors:** Anantharaj Alaganathan, Udhayakumar Gunasekaran, Gurulakshmi Mariappan, Rajkamal Natarajan, Ramkumar Vanaraj, Gokulakumar Balakrishnan

**Affiliations:** 1 PG & Research Department of Physics, Thiru. A. Govindasamy Government Arts College, Tindivanam 604307, Tamil Nadu, India; ananthmatrix84@gmail.com (A.A.);; 2 Department of Chemistry, New Prince Shri Bhavani College of Engineering and Technology, Chennai 600073, Tamil Nadu, India; gurulakshmi.m@gmail.com; 3 Department of Molecular Analytics, Saveetha School of Engineering, Saveetha Institute of Medical and Technical Sciences, Saveetha University, Thandalam, Chennai 602105, Tamil Nadu, India; 4 School of Chemical Engineering, Yeungnam University, Gyeongsan 38541, Republic of Korea

**Keywords:** biogenic nanomaterials, bioactive ZnO nanoparticles, green synthesis, *Leucas aspera*, cancer nanomedicine

## Abstract

Biogenic and bioactive nanomaterials synthesized through green approaches have gained considerable attention for therapeutic applications. In this study, zinc oxide nanoparticles (ZnO NPs) were successfully synthesized using an aqueous leaf extract of *Leucas aspera* as a natural reducing and capping agent to produce phytochemical-functionalized ZnO nanoparticles (LA-ZnO). The synthesized nanoparticles were characterized using UV-Vis, FTIR, XRD, SEM, EDX, and DLS analyses. UV-Vis spectra showed absorption peaks at 391 nm for ZnO and 313 nm for LA-ZnO, while XRD confirmed a hexagonal wurtzite structure with average crystallite sizes of 50.19 and 33.72 nm, respectively. SEM analysis revealed rod-like ZnO and flower-like LA-ZnO morphologies, and EDX confirmed Zn and O with trace carbon from phytochemical surface functionalization. DLS measurements indicated effective colloidal stabilization of LA-ZnO nanoparticles. The bioactive LA-ZnO exhibited concentration-dependent antibacterial activity against Gram-positive and Gram-negative bacteria, showing maximum inhibition against *Staphylococcus aureus* (18 mm at 2000 µg). In vitro MTT assays demonstrated cytotoxicity toward A549 and MCF-7 cancer cells with IC_50_ values of 22.55 and 81.15 µg/mL, respectively. These findings highlight *Leucas aspera*-mediated ZnO nanoparticles as promising biogenic nanomaterials for antimicrobial and therapeutic biomedical applications.

## 1. Introduction

Nanotechnology has revolutionized materials science by enabling the design of materials with unique physicochemical properties at the nanoscale, including high surface area, tunable electronic structures, and enhanced reactivity [1,2,3]. Among various nanomaterials, metal oxide nanoparticles have attracted considerable attention because of their chemical stability, cost-effectiveness, and broad applicability in environmental, technological, and biomedical fields [4,5,6]. Zinc oxide (ZnO) nanoparticles are particularly important owing to their wide direct band gap (3.37 eV), high exciton binding energy, excellent biocompatibility, photocatalytic activity, and antimicrobial properties [7,8,9]. These characteristics have enabled their application in sensors, photocatalysis, environmental remediation, drug delivery, and biomedical technologies [10,11,12]. The physicochemical properties of ZnO nanoparticles are strongly influenced by their size, morphology, crystallinity, and surface characteristics. Various nanostructures, including nanospheres, nanorods, nanoflowers, nanosheets, and hierarchical architectures, have been successfully synthesized and investigated for diverse applications [13,14,15]. Differences in structural features can substantially affect optical absorption, surface reactivity, charge transport, and biological interactions [16,17,18]. In particular, nanoparticles with smaller dimensions exhibit a higher surface-to-volume ratio, which increases the availability of active sites and enhances interactions with surrounding biological systems [19,20,21]. Furthermore, morphology-dependent variations in electron transfer and reactive oxygen species (ROS) generation have been reported to influence the antimicrobial and anticancer performance of ZnO nanomaterials [22,23,24]. Therefore, controlling nanoparticle morphology and surface properties remains an important strategy for optimizing the functional performance of ZnO-based nanomaterials [25,26,27]. Among the various green synthesis approaches, plant-mediated synthesis has emerged as one of the most attractive alternatives due to its simplicity, sustainability, and cost-effectiveness [28,29,30]. Plant extracts contain a complex mixture of bioactive phytochemicals, including flavonoids, phenolic acids, alkaloids, terpenoids, tannins, and glycosides, which participate in nanoparticle formation [31,32,33]. These naturally occurring compounds can simultaneously act as reducing, capping, and stabilizing agents during the synthesis process [34,35,36]. Consequently, the use of plant extracts eliminates the need for additional hazardous chemicals and minimizes environmental impact [37,38,39]. Compared with microbial synthesis, plant-based methods generally require less stringent culture conditions and shorter processing times, making them more suitable for large-scale production [40,41,42]. Moreover, phytochemical-functionalized nanoparticles often exhibit improved colloidal stability and enhanced biological activity due to the presence of bioactive molecules on their surfaces [43,44,45].

Recent investigations have demonstrated that plant-derived ZnO nanoparticles possess significant potential for biomedical applications, particularly in antimicrobial and anticancer therapies [46,47,48]. The antibacterial activity of ZnO nanoparticles is mainly attributed to ROS generation, disruption of bacterial membrane integrity, and the release of Zn^2+^ ions, which collectively impair essential cellular functions [49,50,51]. These mechanisms enable ZnO nanoparticles to effectively inhibit both Gram-positive and Gram-negative bacterial pathogens [52,53,54]. In cancer research, ZnO nanoparticles have attracted considerable attention as potential anticancer agents owing to their ability to induce oxidative stress, mitochondrial dysfunction, and apoptosis in cancer cells [55,56,57]. Elevated intracellular ROS levels can trigger mitochondrial dysfunction, DNA damage, and activation of apoptotic signalling pathways, ultimately leading to cancer cell death [58,59,60]. Furthermore, surface-associated phytochemicals derived from medicinal plants may enhance nanoparticle–cell interactions and contribute synergistically to their therapeutic efficacy [61,62,63]. *Leucas aspera* is a medicinal herb belonging to the family *Lamiaceae* and has been extensively utilized in traditional medicine systems for the treatment of fever, inflammation, skin diseases, and respiratory disorders [64,65,66]. Phytochemical analyses have revealed the presence of numerous biologically active constituents, including flavonoids, phenolics, terpenoids, alkaloids, and tannins [67,68,69]. These compounds exhibit antioxidant, antimicrobial, anti-inflammatory, and cytoprotective properties that are relevant to biomedical applications [70,71,72]. The rich phytochemical profile of *L. aspera* makes it a promising biological resource for the green synthesis of functionalized nanomaterials [73,74,75]. Despite its recognized medicinal importance, studies exploring the synthesis of ZnO nanoparticles using *L. aspera* leaf extract and evaluating their multifunctional biological activities remain relatively limited [76,77,78]. Therefore, further investigation is warranted to understand the potential of *L. aspera*-mediated ZnO nanoparticles as sustainable and effective nanomaterials for biomedical applications.

ZnO nanoparticles have demonstrated significant potential in both antimicrobial and anticancer applications [79,80]. Their antibacterial activity is primarily associated with reactive oxygen species (ROS) generation, Zn^2+^ ion release, and disruption of microbial cell membranes, resulting in effective inhibition of both Gram-positive and Gram-negative bacteria [81,82,83]. In addition, ZnO nanoparticles can induce oxidative stress, mitochondrial dysfunction, apoptosis, and DNA damage in cancer cells, making them promising candidates for cancer therapy [57,58,59,60,61,62,63,64,65]. Surface functionalization through plant-derived phytochemicals may further enhance these biological activities by improving nanoparticle–cell interactions and biocompatibility. Therefore, the present study reports the green synthesis of phytochemical-functionalized ZnO nanoparticles using aqueous leaf extract of *Leucas aspera*. The synthesized nanoparticles were characterized using UV–Vis spectroscopy, FTIR, XRD, SEM, EDX, and DLS analyses to evaluate their optical, structural, morphological, elemental, and colloidal properties. Their antibacterial activity was investigated against Gram-positive (*Bacillus subtilis* and *Staphylococcus aureus*) and Gram-negative (*Escherichia coli* and *Pseudomonas aeruginosa*) bacterial strains, while their anticancer potential was assessed against A549 lung adenocarcinoma and MCF-7 breast cancer cell lines. This work highlights the potential of *L. aspera*-derived ZnO nanoparticles as sustainable and multifunctional nanomaterials for biomedical applications.

## 2. Experimental

### 2.1. Plant Material and Reagents

Fresh leaves of *Leucas aspera* were collected from Thaiyur Village (Viluppuram District, Tamil Nadu, India) and authenticated in the Department of Botany. The leaves were washed with deionized water, air-dried at room temperature, powdered, and stored. Zinc nitrate hexahydrate (Zn(NO_3_)_2_·6H_2_O) and NaOH (both analytical grade, Merck, Darmstadt, Germany) and deionized water were used as received.

### 2.2. Green Synthesis of LA-ZnO

**Figure 1:**
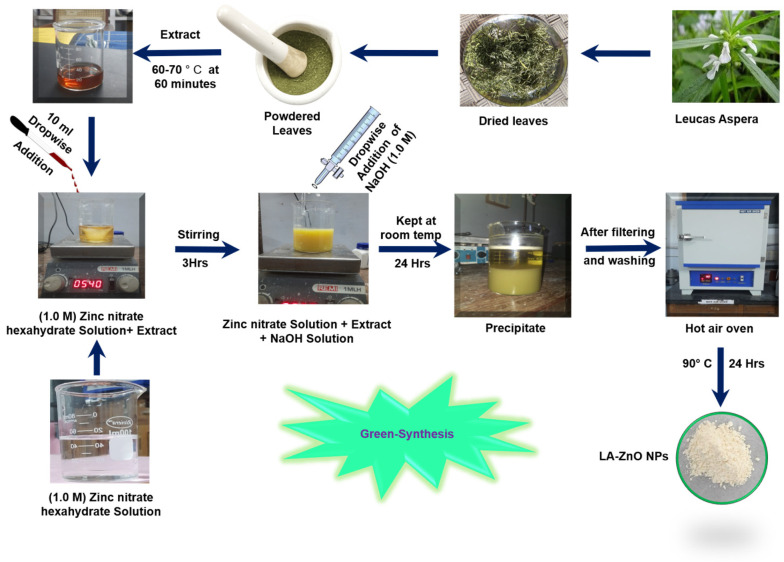
Schematic representation of green synthesized ZnO nanoparticles from *Leucas aspera* leaf extract (LA-ZnO).

Leaf powder (10 g) was boiled with deionized water (100 mL) at 60–70 °C for 60 min, cooled, and filtered through Whatman No. 1 paper. The filtrate was stored at 4 °C and used as *L. aspera* extract. The Zn(NO_3_)_2_·6H_2_O (1.0 M, 100 mL) and NaOH (1.0 M, 100 mL) aqueous solutions were prepared separately. The NaOH solution was added dropwise to the vigorously stirred Zn(NO_3_)_2_ solution at room temperature and stirring was continued for 3 h. The resulting white precipitate was aged for 24 h, filtered, washed with deionized water (3×) and ethanol (2×), dried at 90 °C for 12 h, ground, and stored as ZnO powder. The Zn(NO_3_)_2_·6H_2_O solution (1.0 M, 100 mL) was mixed with *L. aspera* extract (10 mL) and stirred for 1 h. NaOH (1.0 M, 100 mL) was then added dropwise under vigorous stirring until a yellowish-white suspension formed; stirring was continued for 2 h followed by 24 h ageing. The precipitate was collected by filtration, washed with deionized water (3×) and ethanol (2×), dried at 90 °C for 12 h, ground, and stored as LA–ZnO (Figure 1).

### 2.3. Characterization

UV-Vis spectra (200–800 nm) were recorded on a PerkinElmer Lambda 35 spectrophotometer (PerkinElmer Inc., Waltham, MA, USA) using aqueous nanoparticle dispersions (~1 mg mL^−1^). FTIR spectra (400–4000 cm^−1^, KBr pellets) were obtained on a PerkinElmer Spectrum RX I instrument (PerkinElmer Inc., Waltham, MA, USA). Powder XRD patterns were measured on a Bruker D8 Advance diffractometer (Bruker AXS GmbH, Karlsruhe, Germany) with Cu Kα radiation (λ = 0.15406 nm) over 2θ = 10–80°. Crystallite sizes were estimated using the Debye–Scherrer equation. Morphology and elemental composition were examined by SEM–EDX (ZEISS EVO 18; Carl Zeiss AG, Oberkochen, Germany). Hydrodynamic size and polydispersity were determined by DLS (Malvern Instruments Ltd., Malvern, Worcestershire, UK) using aqueous dispersions.

### 2.4. Antibacterial Assay

Antibacterial activity was evaluated by the disc diffusion method against *E. coli*, *P. aeruginosa*, *B. subtilis* and *S. aureus*. Briefly, standardized bacterial suspensions were spread on nutrient agar plates, and sterile discs loaded with ZnO or LA–ZnO (500, 1000, or 2000 µg per disc in DMSO) were placed on the surface. Streptomycin (20 µg per disc) and DMSO served as positive and negative controls, respectively. Plates were incubated at 37 °C for 24 h and inhibition zones were measured, all antibacterial assays were performed under dark conditions to minimize any potential influence of light-induced photocatalytic activity of ZnO nanoparticles.

### 2.5. Cytotoxicity (MTT Assay)

The cytotoxic effects of LA–ZnO were assessed on A549 and MCF-7 cell lines cultured in DMEM supplemented with 10% FBS and antibiotics at 37 °C in 5% CO_2_. Cells (~10^3^ per well) were seeded in 96-well plates, allowed to adhere for 24 h, and then treated with LA–ZnO (6.25–100 µg mL^−1^) or reference drugs for 24 h. After incubation with MTT solution, the formed formazan was dissolved in DMSO and absorbance at 570 nm was recorded. Cell viability (%) was calculated relative to untreated controls and IC_50_ values were obtained from dose response curves. MCF-7 (human breast adenocarcinoma) and A549 (human lung adenocarcinoma) cell lines were selected because they are well-characterized and widely used in anticancer screening studies. These cell lines represent two major cancer types and provide reliable in vitro models for evaluating the cytotoxic effects of LA-ZnO nanoparticles.

### 2.6. Statistical Analysis

All experiments were conducted in triplicate to ensure the reproducibility and reliability of the results. The obtained data are presented as mean ± standard deviation (SD). The consistency of the measurements among independent experiments was evaluated through comparison of the replicate values. Due to the exploratory nature of the present study, formal statistical significance tests were not performed.

## 3. Results and Discussion

### 3.1. UV-Visible Spectroscopy Analysis

**Figure 2:**
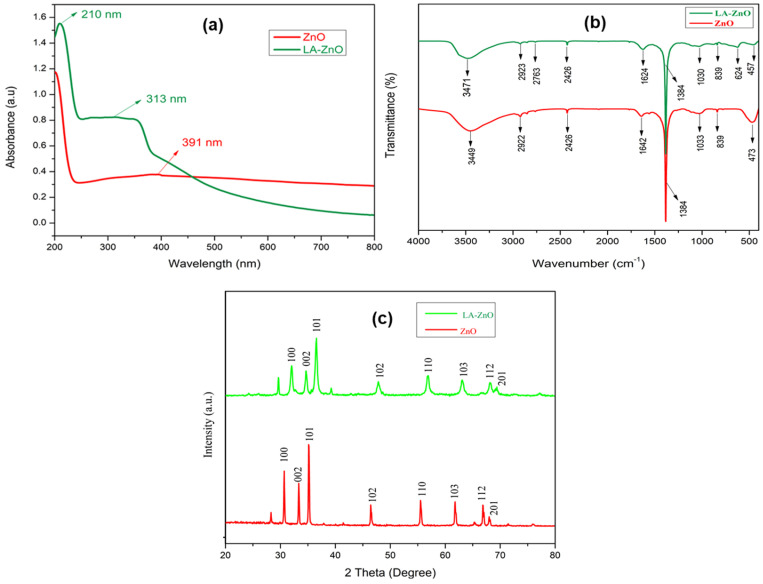
(**a**) UV-Visible spectrum of ZnO and *Leucas aspera*–assisted ZnO nanoparticles (LA-ZnO), (**b**) FTIR spectrums of ZnO and ZnO with “*Leucas aspera*” and (**c**) Powder XRD pattern of ZnO and ZnO with *Leucas aspera* (LA-ZnO).

UV-Visible absorption spectroscopy provides fundamental information regarding nanoparticle formation, size quantization, and bandgap energy characteristics through analysis of characteristic absorption peaks in the ultraviolet and visible wavelength ranges. Figure 2a displays the UV-Visible absorption spectra of pure ZnO and *Leucas aspera*-mediated ZnO nanoparticles (LA-ZnO). The pure ZnO nanoparticles exhibited a prominent and characteristic absorption peak at 391 nm, which corresponds to the intrinsic bandgap transition (π → π* transition) of zinc oxide, confirming successful synthesis of ZnO nanoparticles and validating the hexagonal wurtzite crystalline structure [65]. In contrast, the biosynthesized *Leucas aspera*-mediated ZnO nanoparticles (LA-ZnO) demonstrated a distinctive absorption peak at approximately 313 nm, accompanied by a secondary absorption band at 210 nm. This substantial blue shift in the absorption edge (from 391 nm to 313 nm, representing a hypsochromic shift of approximately 78 nm) indicates a significant reduction in particle size and modification of the particle morphology compared to conventional chemically synthesized ZnO nanoparticles. The observed blue shift in absorption wavelength for LA-ZnO nanoparticles is consistent with established quantum confinement effects, wherein smaller nanoparticles with reduced dimensions exhibit increased bandgap energy and correspondingly shorter wavelength absorption maxima [65]. The phytochemical compounds from the *Leucas aspera* leaf extract, particularly phenolic and flavonoid compounds, appear to facilitate the nucleation of smaller, more uniformly distributed nanoparticles through effective capping and stabilization mechanisms.

The bandgap energy was calculated from the absorption spectra utilizing the Tauc plot methodology, employing the following relationship:

(αhν)^(1/2)^ = K(hν − E_g_)

where α represents the absorption coefficient, h represents Planck’s constant (6.626 × 10^−34^ J·s), ν represents the frequency of incident radiation, K represents a proportionality constant, and E_g_ represents the bandgap energy. The bandgap energy values were determined to be 3.17 eV for pure ZnO and 3.96 eV for LA-ZnO nanoparticles, respectively. The significantly higher bandgap energy (0.79 eV difference) for LA-ZnO compared to pure ZnO confirms the substantially reduced particle size of the phytochemical-mediated nanoparticles. These findings conclusively demonstrate that *Leucas aspera* leaf extract functions effectively as both a reducing agent (converting Zn^2+^ cations to Zn^0^ and subsequently to ZnO) and a stabilizing/capping agent (preventing uncontrolled particle growth and aggregation) during the synthesis process. The phytochemical compounds present in the leaf extract appear to preferentially stabilize smaller nanoparticle dimensions through surface coordination, resulting in the enhanced bandgap energy characteristic of quantum-confined nanomaterials.

Fourier transform infrared (FTIR) spectroscopy was employed to investigate the functional groups present in the synthesized nanoparticles and to examine the possible interactions between plant-derived biomolecules and the ZnO nanoparticle surface (Table 1). FTIR analysis is a useful tool for identifying characteristic functional groups that may participate in the reduction, stabilization, and capping of biosynthesized nanoparticles. The FTIR spectra of pure ZnO and *Leucas aspera*-mediated ZnO (LA–ZnO) nanoparticles are presented in Figure 2b. For pure ZnO, characteristic absorption bands were observed at 3449 cm^−1^, corresponding to O–H stretching vibrations of surface hydroxyl groups and adsorbed water molecules, 2922 cm^−1^ assigned to C–H stretching vibrations of residual organic species, 2426 cm^−1^ associated with adsorbed atmospheric species, 1642 cm^−1^ attributed to C=O stretching vibrations and/or bending vibrations of adsorbed water molecules, 1384 cm^−1^ corresponding to C–N stretching or symmetric stretching vibrations of surface-bound species, 1033 cm^−1^ assigned to C–O stretching vibrations, 839 cm^−1^ related to out-of-plane C–H bending vibrations, and 473 cm^−1^ corresponding to the characteristic Zn–O stretching vibration, confirming the formation of ZnO nanoparticles. The FTIR spectrum of LA–ZnO nanoparticles exhibited absorption bands at 3471 cm^−1^, 2923 cm^−1^, 2763 cm^−1^, 2426 cm^−1^, 1624 cm^−1^, 1384 cm^−1^, 1030 cm^−1^, 839 cm^−1^, 624 cm^−1^, and 457 cm^−1^. The broad band at 3471 cm^−1^ is attributed to O–H stretching vibrations and may originate from hydroxyl-containing biomolecules present in the plant extract. The bands at 2923 and 2763 cm^−1^ are associated with C–H stretching vibrations of aliphatic groups, while the absorption band at 1624 cm^−1^ may be attributed to C=O stretching and/or amide-associated functional groups. The bands at 1384 and 1030 cm^−1^ are consistent with C–N and C–O stretching vibrations, respectively, which may arise from plant-derived organic constituents adsorbed on the nanoparticle surface. The bands observed at 624 and 457 cm^−1^ are assigned to Zn–O vibrational modes, confirming the formation of ZnO nanoparticles. Compared with pure ZnO, slight shifts in band positions and the appearance of additional absorption features were observed in the LA–ZnO spectrum. These changes suggest interactions between ZnO nanoparticles and biomolecules derived from the *Leucas aspera* extract. The shift in the broad O–H stretching band and the presence of bands associated with oxygen- and nitrogen-containing functional groups indicate that phytochemical constituents containing hydroxyl, carbonyl, and related functionalities may have contributed to nanoparticle formation and stabilization. Such biomolecules can facilitate the reduction process and subsequently adsorb onto the nanoparticle surface, providing steric and/or electrostatic stabilization. It should be noted that FTIR analysis provides information regarding functional groups but does not allow definitive identification of specific phytochemical compounds. Therefore, the assignments presented here are based on characteristic functional-group vibrations and their reported occurrence in plant-derived biomolecules. Further phytochemical characterization using complementary analytical techniques such as LC–MS, GC–MS, or NMR would be required for definitive compound identification.

### 3.2. X-Ray Diffraction (XRD) Analysis

**Figure 3:**
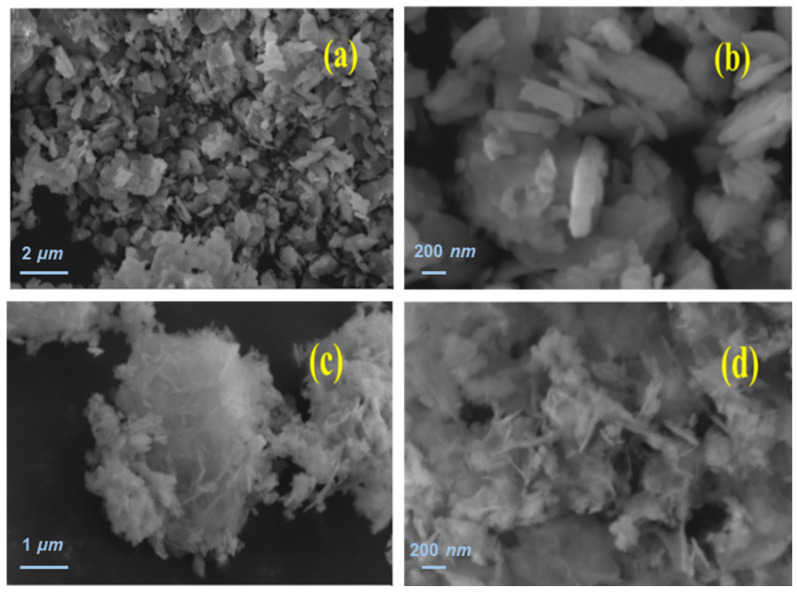
SEM micrograph of (**a**,**b**) ZnO and (**c**,**d**) biosynthesized ZnO with *L. aspera* leaf extract (LA-ZnO) nanoparticles.

X-ray diffraction (XRD) analysis was used to determine the crystalline structure, phase purity, and crystallite size of the synthesized nanoparticles. The powder XRD patterns of pure ZnO and *Leucas aspera* mediated ZnO (LA–ZnO) are shown in Figure 3c and exhibit sharp, well-defined reflections characteristic of highly crystalline materials, indicating good crystallinity and low structural disorder. For pure ZnO, the main diffraction peaks appear at 2θ values of 31.69°, 34.36°, 36.17°, 47.47°, 56.51°, 62.77°, 66.28°, 67.84°, and 69.97°, whereas LA–ZnO shows corresponding reflections at 31.04°, 34.68°, 36.52°, 47.81°, 56.75°, 62.05°, 66.67°, 67.18°, and 69.29°. All these peaks can be indexed to the (100), (002), (101), (102), (110), (103), (200), (112), and (201) planes of hexagonal wurtzite ZnO, in excellent agreement with JCPDS card no. 36-1451, confirming that both samples crystallize in the typical wurtzite structure of zinc oxide. The absence of additional reflections attributable to other crystalline phases or impurities demonstrates high phase purity and the lack of secondary oxide phases.

The average crystallite size of the nanoparticles was estimated from the diffraction data using the Debye-Scherrer equation, D = kλ/(βcosθ), applied to the most intense (002) reflection, where k = 0.89 is the shape factor, λ = 0.15406 nm is the Cu Kα wavelength, β is the full width at half maximum in radians, and θ is the Bragg angle. Using this approach, the average crystallite size of pure ZnO was found to be 50.19 ± 2.3 nm, while that of LA–ZnO decreased to 33.72 ± 1.8 nm, corresponding to an approximate 33% reduction in crystallite size. This marked size reduction is attributed to the nucleation-controlling and growth-inhibiting role of *Leucas aspera* phytochemicals, particularly phenolic and flavonoid constituents, which adsorb on growing crystallite surfaces and limit further growth via steric hindrance and surface passivation. The smaller crystallite size of LA–ZnO is consistent with the higher optical bandgap (3.96 eV versus 3.17 eV for pure ZnO) obtained from UV–Vis measurements, supporting the presence of enhanced quantum confinement effects in the green-synthesized nanoparticles. Moreover, the reduced crystallite dimensions result in an increased surface-area-to-volume ratio, which is expected to improve surface reactivity and contribute to the enhanced antibacterial and anticancer performance observed for LA–ZnO. Detailed XRD parameters, including peak positions, FWHM, d-spacings, and individual crystallite size values, are summarized in Table 2.

### 3.3. SEM with EDAX Analysis

Scanning electron microscopy (SEM) combined with energy-dispersive X-ray (EDAX) spectroscopy was employed to examine the surface morphology and elemental composition of the synthesized nanoparticles. Representative SEM micrographs of pure ZnO and *Leucas aspera*-mediated ZnO (LA-ZnO) nanoparticles are shown in Figure 3 at magnifications of 50,000× to 100,000×. Pure ZnO nanoparticles (Figure 3a,b) exhibit predominantly rod-shaped or cylindrical morphologies with aspect ratios of 2:1 to 5:1 (length 60–150 nm, diameter 20–40 nm), featuring sharp edges indicative of high crystallinity and minimal surface defects, with particles observed primarily as isolated crystallites or small loose aggregates. In marked contrast, LA-ZnO nanoparticles (Figure 3c,d) display distinctive flower-like hierarchical architectures with overall diameters of 150–400 nm, composed of thin plate-like petals (10–30 nm thick) radiating from central nucleation sites; these subunits appear single-crystalline and exhibit moderate to substantial aggregation into larger secondary structures, reflecting phytochemical-directed anisotropic growth and controlled assembly along specific crystallographic planes. The flower-like morphology observed for the LA-ZnO nanoparticles is of particular biomedical interest, as hierarchical nanostructures with high surface area may facilitate enhanced cellular interactions and improve biological activity, thereby contributing to their antibacterial and anticancer potential.

**Figure 4:**
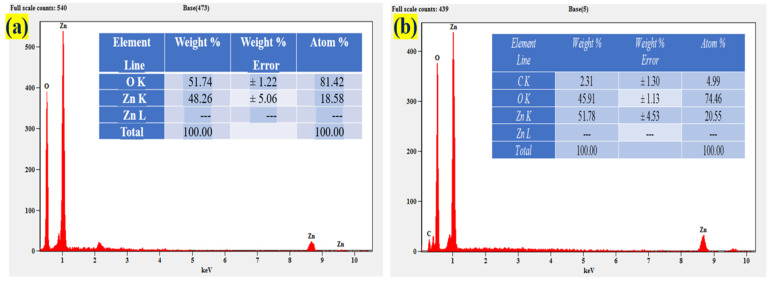
EDAX images illustrating the chemical element and purity of (**a**) ZnO and (**b**) ZnO with *L. aspera* leaf extract (LA-ZnO) nanoparticles.

EDAX analysis (Figure 4a,b) confirmed the elemental purity and composition of both samples. Pure ZnO showed Zn (48.26 ± 1.5 wt%, 42.8 at%) and O (51.74 ± 1.8 wt%, 57.2 at%), closely matching the theoretical ZnO stoichiometry (Zn 48.6 wt%, O 51.4 wt%) and indicating phase-pure formation without significant oxygen vacancies. For LA-ZnO, the composition was Zn (51.78 ± 2.1 wt%, 44.2 at%), O (45.91 ± 1.6 wt%, 50.1 at%), and trace C (2.31 ± 0.8 wt%, 5.7 at%), with the detected carbon unambiguously attributable to residual *L. aspera* phytochemicals (polysaccharides, phenolics, proteins) incorporated during green synthesis; the slight Zn enrichment and O deficiency further supports organic surface modification and potential partial O substitution by carbon-containing moieties.

**Figure 5:**
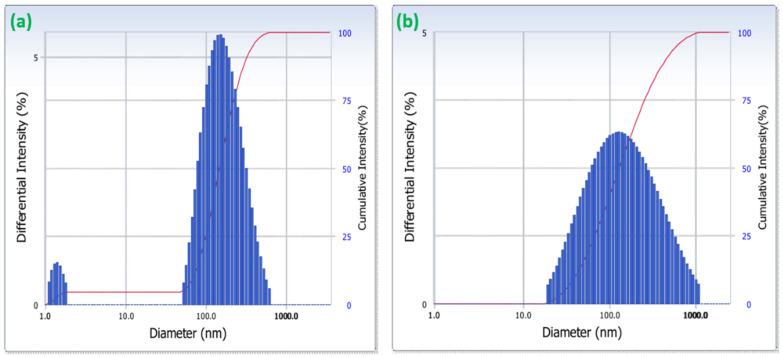
(**a**,**b**) Size distribution profile of LA-ZnO NPs via Dynamic Light Scattering.

Dynamic light scattering (DLS) measurements provided the hydrodynamic size distributions of the nanoparticles in aqueous suspension (Figure 5a,b). Pure ZnO exhibited an average hydrodynamic diameter of 179.2 ± 8.5 nm with a polydispersity index (PDI) of 0.352 (narrow, unimodal distribution), while LA-ZnO showed 206.8 ± 11.2 nm with PDI 0.400 (moderately polydisperse, near-unimodal). These hydrodynamic sizes substantially exceed the XRD-derived crystallite sizes (50.19 nm for ZnO, 33.72 nm for LA-ZnO), a common observation attributable to hydration shells and associated counterions in solution, where D DLS ~ D XRD + 2 × (shell thickness) and shell thicknesses of 60–90 nm are consistent with the data. The larger apparent size and modestly increased PDI of LA-ZnO, despite its smaller crystalline core, directly reflect the extended organic corona from phytochemical capping, which enhances colloidal stability while promoting morphological diversity. Both PDI values (<0.5) confirm suitable monodispersity for biomedical use. Collectively, SEM, EDAX, and DLS analyses demonstrate that *L. aspera* extract not only reduces Zn^2+^ to ZnO but also templates unique flower-like architectures with organic-functionalized surfaces that augment stability, dispersibility, and biointerface properties.

### 3.4. Antibacterial Activity Analysis

**Figure 6:**
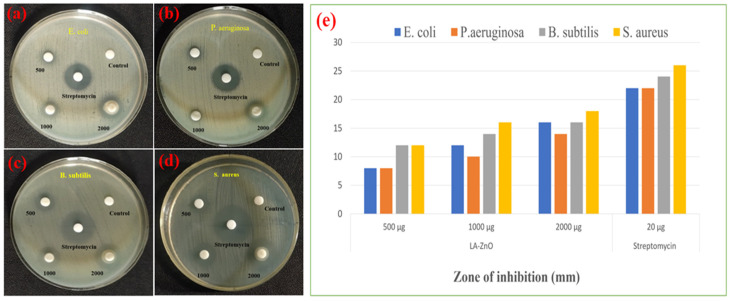
Visual evidence of LA-ZnO nanoparticles’ antibacterial activity against various bacterial strains: (**a**) *E. coli*, (**b**) *P. aeruginosa*, (**c**) *B. subtilis*, (**d**) *S. aureus* through photographic image, Antibacterial assessment of LA-ZnO NPs using the inhibition zone method on different bacterial strains (**e**).

Zinc oxide nanoparticles represent promising alternatives to conventional antibiotics amid rising multidrug resistance, acting through multifaceted mechanisms including reactive oxygen species (ROS) generation, Zn^2+^ ion release, direct cell wall disruption, and metabolic interference. The antibacterial efficacy of *Leucas aspera*-mediated ZnO nanoparticles (LA-ZnO) was evaluated against Gram-negative (*Escherichia coli*, *Pseudomonas aeruginosa*) and Gram-positive (*Bacillus subtilis*, *Staphylococcus aureus*) strains using the disc diffusion method at concentrations of 500, 1000, and 2000 µg per disc, with streptomycin (20 µg) as the positive control (Figure 6a–e). *E. coli* showed moderate inhibition zones of 8, 12, and 16 mm, while *P. aeruginosa* exhibited slightly lower susceptibility at 8, 12, and 14 mm. Gram-positive strains demonstrated markedly higher sensitivity: *B. subtilis* produced zones of 12, 14, and 16 mm, and *S. aureus* was most susceptible with 12, 16, and 18 mm inhibition zones, approaching streptomycin’s 26 mm benchmark. This pronounced differential activity reflects fundamental differences in cell wall architecture. Gram-positive bacteria possess thick (50–100 nm), porous peptidoglycan layers that permit penetration of LA-ZnO nanoparticles (33.72 nm crystallites), enabling direct mechanical disruption, cytoplasmic membrane breaching, and intracellular content leakage leading to lysis. In contrast, Gram-negative bacteria are protected by a thin peptidoglycan layer overlaid with a lipopolysaccharide (LPS) outer membrane that acts as a permeability barrier, substantially restricting nanoparticle access and requiring prolonged ROS-mediated lipid peroxidation or localized membrane breaches for activity. These observations align with extensive literature demonstrating superior ZnO efficacy against Gram-positive strains.

Multiple synergistic mechanisms underpin LA-ZnO antibacterial action. Photocatalytic ROS generation (O_2_^−^-, -OH, H_2_O_2_) damages DNA, peroxidizes membrane lipids, and oxidizes proteins even without external illumination. ZnO dissolution releases toxic Zn^2+^ ions that disrupt enzyme cofactors, respiration, DNA replication, and protein synthesis. Direct nanoparticle–cell wall interactions exploit electrostatic attraction between positively charged ZnO surfaces and negatively charged bacterial envelopes, generating local stress and membrane potential collapse. Cellular internalization further amplifies intracellular damage to ribosomes and metabolic pathways. The observed concentration dependence reflects cumulative damage accumulation. LA-ZnO exhibits equivalent or enhanced activity relative to pure ZnO, attributable to its smaller crystallite size (33.72 vs. 50.19 nm) yielding higher surface-area-to-volume ratios and ROS production, phytochemical surface modification enhancing cell interactions, and additive antimicrobial effects from adsorbed phenolics, flavonoids, and alkaloids. The exceptional potency against *S. aureus* (18 mm zone), a major nosocomial pathogen including MRSA strains, underscores LA-ZnO’s clinical potential. Quantitative inhibition zone data are summarized in Table 3. Streptomycin was used as a positive reference control to confirm bacterial susceptibility. However, direct quantitative comparison between inhibition zones produced by LA-ZnO nanoparticles and streptomycin should be interpreted with caution because the nanoparticles were tested at substantially higher concentrations (500–2000 µg/disc) than the antibiotic control (20 µg/disc). In addition, nanoparticles and conventional antibiotics differ considerably in their physicochemical properties, diffusion behaviour in agar media, and mechanisms of antimicrobial action.

### 3.5. Anticancer Activity

Cancer remains one of the leading global health threats, with lung and breast malignancies accounting for a substantial proportion of new cancer cases and cancer-related deaths worldwide, underscoring the urgent need for effective therapeutic strategies. Zinc oxide nanoparticles have attracted considerable attention as potential anticancer agents due to their ability to induce oxidative stress, mitochondrial dysfunction, DNA damage, and apoptosis in cancer cells [81,82,83]. The anticancer potential of *Leucas aspera*-mediated ZnO nanoparticles (LA-ZnO) was assessed against A549 (lung adenocarcinoma) and MCF-7 (breast adenocarcinoma) cell lines using the MTT assay at concentrations of 6.25–100 µg mL^−1^, with cisplatin and 5-fluorouracil as references for A549 and MCF-7, respectively. Although conventional chemotherapeutic agents remain the cornerstone of cancer treatment, nanoparticle-based systems have attracted considerable interest because of their unique physicochemical properties, including high surface area and enhanced interactions with biological targets. The LA-ZnO nanoparticles investigated in this study demonstrated in vitro anticancer activity; however, direct comparisons with established therapeutic modalities require further studies involving normal cell lines, mechanistic investigations, and in vivo efficacy and safety assessments.

**Figure 7:**
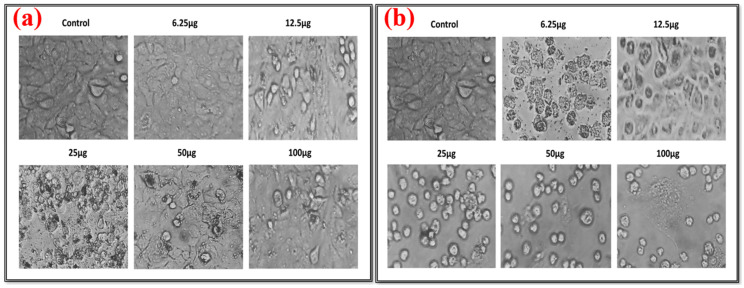
Microscopic images of A549 lung cancer cells treated with (**a**) LA-ZnO nanoparticles and (**b**) Cisplatin (reference standard) at different concentrations (6.25, 12.5, 25, 50, and 100 µg/mL) compared with the untreated control. Images were acquired using an inverted phase-contrast microscope at 200× magnification. Scale bar ~100 µm.

**Figure 8:**
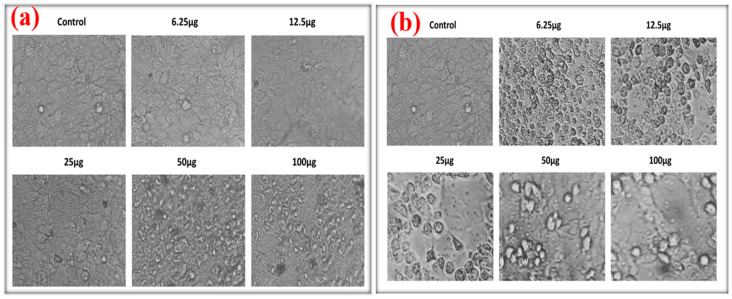
Morphological changes in (**a**) MCF-7 and (**b**) A549 cells following treatment with biosynthesized AgNPs at different concentrations (6.25–100 μg/mL) for 24 h. Images were captured using an inverted phase-contrast microscope at 200× magnification. Scale ~100 μm.

**Figure 9:**
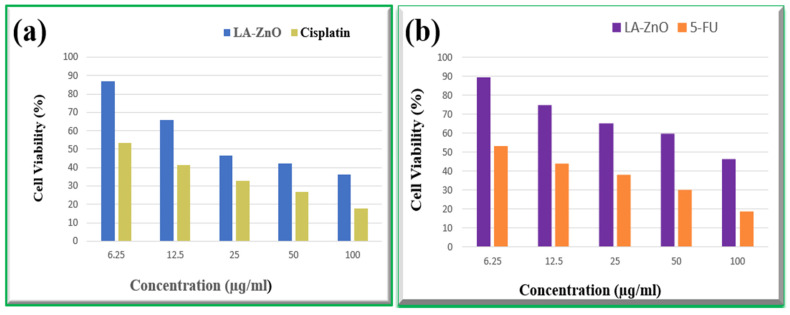
Cell viability (%) vs. concentration (**a**) compares LA-ZnO NPs and cisplatin (reference standard) on A549 (**b**) compares LA-ZnO NPs and 5F-U (reference standard) on the MCF-7 cell line.

**Figure 10:**
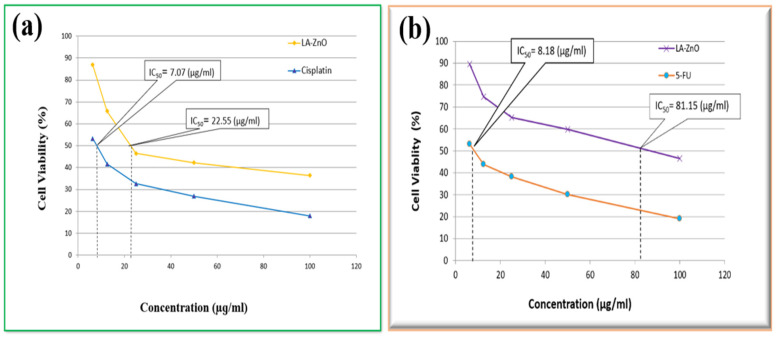
The IC_50_ value and decay curves of A549 cell survival viability (**a**) and MCF-7 cells (**b**), compared with reference standard at different viability concentration.

The SEM micrographs shown represent the morphology of the synthesized LA-ZnO nanoparticles at the time of characterization and correspond to the same nanoparticle batch used throughout the study. Microscopic examination of treated cells revealed concentration-dependent morphological hallmarks of apoptosis (Figure 7a,b and Figure 8a,b). Untreated control cells displayed intact nuclei, extended filopodia, lamellipodia, and strong adherence with robust monolayer formation. At low doses (6.25 µg mL^−1^), minimal changes were evident. Intermediate concentrations (12.5–25 µg mL^−1^) induced cell rounding, process retraction, shrinkage, chromatin condensation, and partial detachment. High doses (50–100 µg mL^−1^) caused severe rounding, nuclear fragmentation (karyorrhexis), membrane blebbing, apoptotic body formation, and extensive detachment, consistent with caspase activation and mitochondrial-mediated apoptosis. MTT dose–response analysis quantified cell viability through mitochondrial dehydrogenase activity (Figure 9a,b and Figure 10a,b). Against A549 cells, LA-ZnO nanoparticles exhibited concentration-dependent cytotoxic activity against A549 and MCF-7 cells, with IC_50_ values of 22.55 ± 1.8 and 81.15 ± 2.3 µg mL^−1^, respectively (Table 4 and Table 5). Although the observed activity against A549 cells was within the same order of magnitude as that of the reference drug cisplatin under the present experimental conditions, such comparisons should be interpreted cautiously because nanoparticles and conventional chemotherapeutic agents differ substantially in their mechanisms of action, cellular uptake, and pharmacological behaviour. Therefore, the current results should be considered as preliminary evidence of anticancer potential rather than proof of therapeutic equivalence. Further investigations involving normal cell lines, mechanistic studies, and in vivo models are necessary to establish the efficacy and safety of LA-ZnO nanoparticles. Untreated cells were used as the negative control and were considered to represent 100% cell viability. The viability of treated cells was calculated relative to the untreated control based on the corresponding MTT absorbance measurements. For MCF-7 cells, LA-ZnO showed moderate activity with an IC_50_ of 81.15 ± 2.3 µg mL^−1^, less potent than 5-fluorouracil (IC_50_ ~8–12 µg mL^−1^; ratio 6.7–10.1×), reflecting MCF-7′s inherent resistance from elevated multidrug efflux pumps (MDR1/P-gp), robust antioxidant defences (SOD, catalase, glutathione peroxidase), and dysfunctional mitochondria. The 3.6-fold higher potency against A549 versus MCF-7 arises from cell-specific vulnerabilities: A549′s high metabolic rate, ROS susceptibility, lower MDR expression, and efficient apoptotic machinery contrast with MCF-7′s protective adaptations. LA-ZnO cytotoxicity involves synergistic mechanisms: (1) ROS overproduction (O_2_^−^-, -OH, H_2_O_2_) causing DNA/protein/lipid damage and mitochondrial collapse; (2) Zn^2+^ release disrupting enzyme cofactors, Ca^2+^ homeostasis, and apoptosis regulators; (3) mitochondrial localization triggering cytochrome c release, caspase-3/9 activation, and PARP cleavage; (4) DNA damage inducing p53-mediated G_2_/M arrest and fragmentation; and (5) phytochemical synergy from adsorbed phenolics/flavonoids providing pro-oxidant effects, ER modulation (relevant for MCF-7), and anti-angiogenic activity. These pathways amplify cancer cell death while potentially minimizing off-target toxicity. Compared to the reference drug cisplatin, LA-ZnO nanoparticles exhibited notable cytotoxic activity against A549 cells, while showing moderate activity against MCF-7 cells. However, direct comparisons should be interpreted with caution because the tested concentrations differed substantially between LA-ZnO nanoparticles and cisplatin. Dose–response curves and IC_50_ values are presented in Figure 10a,b, providing a basis for further investigation of the anticancer potential of LA-ZnO nanoparticles. The antibacterial and anticancer activities observed for the LA-ZnO nanoparticles may be partially attributed to the generation of reactive oxygen species (ROS), which can induce oxidative stress, membrane damage, protein oxidation, and DNA damage in microbial and cancer cells. Previous studies have reported that ZnO nanoparticles are capable of producing ROS, leading to cellular dysfunction and cell death. Nevertheless, direct ROS measurements were not performed in the present study; therefore, the involvement of ROS is proposed as a potential mechanism and warrants further investigation.

The multifunctional properties exhibited by the synthesized LA-ZnO nanoparticles suggest their considerable potential for advanced biomedical and regenerative applications. The strong antibacterial activity observed against both Gram-positive and Gram-negative pathogens indicates that these bioactive nanoparticles may serve as effective antimicrobial agents for wound dressings, implant coatings, and tissue-engineering scaffolds, where prevention of bacterial colonization and biofilm formation is critically important for successful tissue regeneration. In addition, the flower-like hierarchical morphology, reduced crystallite size, and phytochemical-functionalized surface are expected to enhance cell–material interactions, surface reactivity, and biological integration in regenerative environments. The cytotoxicity toward A549 and MCF-7 cancer cells further highlights the potential of LA-ZnO nanoparticles as multifunctional nanotherapeutic platforms for localized cancer treatment. Moreover, the presence of bioactive phytochemical groups on the nanoparticle surface may facilitate future drug immobilization, targeted delivery, and controlled release of therapeutic molecules. The combined inorganic ZnO core and naturally derived organic surface modification provide a biogenic platform capable of integrating antimicrobial, anticancer, and therapeutic delivery functionalities within a single nanostructured system. Therefore, *Leucas aspera*-mediated ZnO nanoparticles represent promising candidates for future applications in tissue regeneration, antimicrobial biomaterials, and targeted nanomedicine.

The biogenic characteristics of the synthesized LA-ZnO nanoparticles arise primarily from the phytochemical-assisted green synthesis process, in which naturally occurring bioactive compounds from *Leucas aspera* function simultaneously as reducing, stabilizing, and surface-functionalizing agents. This bioinspired surface modification generates organic–inorganic hybrid nanostructures with enhanced interfacial interactions toward biological systems, improved colloidal stability, and favourable bioactivity. The distinctive flower-like hierarchical morphology, reduced crystallite size (33.72 nm), and phytochemical-functionalized surface collectively contribute to increased surface reactivity, enhanced cellular interactions, and efficient reactive oxygen species generation, which are highly desirable characteristics for regenerative biomedical applications. Furthermore, the strong antibacterial efficacy against clinically relevant pathogens highlights the potential utility of LA-ZnO nanoparticles in antimicrobial wound dressings, implant coatings, and tissue-engineering scaffolds where prevention of bacterial colonization and biofilm formation is critically important. In addition, the cytotoxicity toward cancer cells demonstrates the capability of these nanoparticles to function as multifunctional therapeutic nanoplatforms for localized cancer treatment and future targeted drug delivery systems. The presence of phytochemical surface groups may further facilitate drug conjugation, controlled release behaviour, and improved bioavailability of therapeutic molecules. Therefore, the present study establishes *Leucas aspera*-mediated ZnO nanoparticles as promising biogenic and bioactive nanomaterials with significant potential for antimicrobial biomaterials, and advanced nanomedicine applications. There are few limitations of the present study should be acknowledged. The antibacterial activity of LA-ZnO nanoparticles was assessed at concentrations substantially higher than that of the streptomycin reference control. Therefore, the observed inhibition zone diameters should be considered indicative of antibacterial activity and not as a direct measure of relative antimicrobial potency. In addition, the long-term colloidal stability and zeta potential of the synthesized LA-ZnO nanoparticles were not investigated. These parameters are important for understanding nanoparticle dispersion behaviour, storage stability, surface charge characteristics, and potential biological interactions. Future studies should therefore include zeta potential measurements, long-term stability assessments, and evaluation under standardized antimicrobial testing conditions to provide a more comprehensive characterization of the nanoparticles and their potential biomedical applications.

## 4. Conclusions

This study successfully demonstrates a biogenic green synthesis approach for the fabrication of bioactive zinc oxide nanoparticles using *Leucas aspera* leaf extract as a natural reducing and surface-functionalizing agent. The phytochemical-mediated LA-ZnO nanoparticles exhibited enhanced physicochemical properties compared with chemically synthesized ZnO, including a reduced crystallite size of 33.72 nm compared to 50.19 nm for pure ZnO, a widened bandgap of 3.96 eV, and distinctive flower-like hierarchical morphologies. FTIR, EDX, and DLS analyses confirmed effective phytochemical capping and colloidal stabilization with a hydrodynamic diameter of 206.8 nm and PDI value of 0.400. The bioactive LA-ZnO nanoparticles demonstrated strong concentration-dependent antibacterial activity against both Gram-positive and Gram-negative bacterial strains, showing a maximum inhibition zone of 18 mm against *Staphylococcus aureus* at 2000 µg. In vitro cytotoxicity studies revealed anticancer activity with IC_50_ values of 22.55 µg/mL for A549 lung cancer cells and 81.15 µg/mL for MCF-7 breast cancer cells, indicating enhanced efficacy toward lung adenocarcinoma cells. The improved biological performance is attributed to reduced particle size, increased surface reactivity, ROS generation, and phytochemical surface functionalization. The novelty of this work lies in the development of a green synthesis route for ZnO nanoparticles using *Leucas aspera* extract and the integrated assessment of their physicochemical, antibacterial, and anticancer properties. The findings demonstrate the potential of plant-mediated ZnO nanoparticles as multifunctional nanomaterials for future biomedical applications. A limitation of the present study is the absence of cytotoxicity evaluation on normal mammalian cell lines. Consequently, although LA-ZnO nanoparticles exhibited significant anticancer activity against A549 and MCF-7 cells, the present findings are limited to in vitro evaluation of cancer cell lines. Further investigations involving normal human cell lines, long-term toxicity studies, and in vivo biocompatibility assessments are necessary to comprehensively evaluate their safety and potential for biomedical applications. These findings highlight *Leucas aspera*-mediated ZnO nanoparticles as promising biogenic and multifunctional nanomaterials for regenerative biomedical applications, antimicrobial coatings, and future targeted drug delivery systems.

## Figures and Tables

**Table 1:** FTIR Spectral Analysis of Synthesized ZnO and LA-ZnO Nanoparticles.

Peak Position (cm^−1^) ZnO	Peak Position (cm^−1^) LA-ZnO	Bond/Functional Group	Assignment
3449	3471	O-H stretching	Surface hydroxyl groups, phenolic OH
2922	2923	C-H stretching	Aliphatic and aromatic C-H
2426	2426	C-H stretching	Alkene/alkyne C-H
-	2763	C-H stretching	Fatty acid/alkane C-H
1642	1624	C=O stretching	Amide II band, conjugated C=O (flavonoids)
1384	1384	C-N stretching	Aromatic compounds, alkaloids
1033	1030	C-O stretching	Polysaccharides, ethers, phenolic C-O
839	839	Aromatic C-H bending	Aromatic ring vibrations
-	624	Zn-O stretching	Modified surface Zn-O
473	457	Zn-O stretching	Primary metal-oxygen vibration

**Table 2:** X-Ray Diffraction Profile and Crystallite Size Analysis.

Peak	2θ (°) ZnO	2θ (°) LA-ZnO	FWHM (°) ZnO	FWHM (°) LA-ZnO	d-Spacing Å ZnO	d-Spacing Å LA-ZnO	hkl	Crystallite Size nm (ZnO)	Crystallite Size nm (LA-ZnO)
1	31.70	31.04	0.1968	0.3542	2.9126	2.7932	100	41.86	23.33
2	34.36	34.68	0.1476	0.2755	2.6859	2.5865	002	56.18	30.20
3	36.18	36.53	0.1968	0.3149	2.5511	2.4599	101	42.34	26.56
4	47.48	47.82	0.1968	0.1574	1.9538	1.9022	102	43.93	55.20
5	56.51	56.76	0.2460	0.1920	1.6554	1.6207	110	36.49	47.02
6	62.77	62.05	0.2460	0.1574	1.5018	1.4744	103	37.63	59.21
7	66.28	66.68	0.1476	0.4723	1.4293	1.4028	200	63.91	20.13
8	67.84	67.19	0.1476	0.3936	1.3997	1.3753	112	64.48	24.37
9	69.98	69.29	0.1476	0.5510	1.3790	1.3560	201	64.91	17.52
Average Crystallite Size (nm)								50.19 ± 2.3	33.72 ± 1.8

**Table 3:** Quantitative Antibacterial Activity Data.

Bacterial Strain	500 µg (mm)	1000 µg (mm)	2000 µg (mm)	Streptomycin 20 µg (mm)
*E. coli*	8 ± 0.5	12 ± 0.6	16 ± 0.8	25 ± 1.0
*P. aeruginosa*	8 ± 0.6	12 ± 0.7	14 ± 0.9	23 ± 0.9
*B. subtilis*	12 ± 0.7	14 ± 0.8	16 ± 0.6	24 ± 1.1
*S. aureus*	12 ± 0.6	16 ± 0.7	18 ± 0.5	26 ± 0.8

**Table 4:** MTT assay results showing concentration-dependent cytotoxicity of LA-ZnO nanoparticles against A549 lung adenocarcinoma cells: percentage cell viability (mean ± SD, *n* = 3), reduction from control, and calculated IC_50_ = 22.55 µg mL^−1^.

Concentration (µg/mL)	% Cell Viability (Mean ± SD)	Reduction from Control (%)
Control (0)	100.0 ± 0.0	-
6.25	82.3 ± 3.2	17.7%
12.5	68.4 ± 2.8	31.6%
25	48.2 ± 3.5	51.8%
50	28.6 ± 2.1	71.4%
100	12.4 ± 1.9	87.6%

**Table 5:** MTT assay results showing concentration-dependent cytotoxicity of LA-ZnO nanoparticles against MCF-7 breast adenocarcinoma cells: percentage cell viability (mean ± SD, *n* = 3), reduction from control, and calculated IC_50_ = 81.15 µg mL^−1^.

Concentration (µg/mL)	% Cell Viability (Mean ± SD)	Reduction from Control (%)
Control (0)	100.0 ± 0.0	-
6.25	88.6 ± 2.9	11.4%
12.5	76.3 ± 3.1	23.7%
25	61.8 ± 2.7	38.2%
50	41.2 ± 2.4	58.8%
100	18.9 ± 2.2	81.1%

## Data Availability

The original contributions presented in this study are included in the article. Further inquiries can be directed to the corresponding authors.
